# Dr. Koch on the Cholera

**Published:** 1884-09

**Authors:** 


### Selections.

### DR. KOCH ON THE CHOLERA.

An important and influential conference* upon cholera was opened in Berlin at the Imperial Board of Health on the evening of July 26th. There were present Drs. v. Bergmann, Coler, Eulenberg, B. Fränkel, Gaffky, Hirsch, Koch, Leyden, S. Neumann, Pistor, Schubert, Skreczka, Struck, Virchow and Wollfhügel. The conference had been called at the instance of the Berlin Medical Society, whose President, Prof. Virchow, explained that it was thought advisable Dr. Koch should, in the first instance, give a demonstration of his work before a smaller body than the whole society, so that the proceedings might be fully reported in the medical press. He mentioned that Herr Director Lucanus and President Sydow had expressed their regret at being unable to be present, as well as many others, including Drs. von Lauer, von Frerichs, Mehlhausen and

* A detailed report is published in the Berliner Klinische Wochenschrift of Aug. 4th.

Kersandt. Before the meeting Dr. Koch exhibited microscopical specimens and drawings of the cholera bacillus, and demonstrated the method of its preparation and cultivation. The preparations included specimens of choleraic dejections dried on covering glasses, stained with fuchsin or methyl-blue, and examined with oil immersion, one-twelfth, and Abbe's condenser; also sections of intestine preserved in absolute alcohol, and stained with methyl-blue. There were also cultures in gelatin, etc.

Dr. Koch commenced by remarking that what was required for the prevention of cholera was a scientific basis. Many and diverse views as to its mode of diffusion and infection prevailed, but they furnished no safe ground for prophylaxis. On the one hand, it was held that cholera is a specific disease originating in India; on the other, that it may arise spontaneously in any country and own no specific cause. One view regards the infection to be conveyed only by the patient and his surroundings; the other that it is spread by merchandise, by healthy individuals, and by atmospheric currents. There is a like discrepancy in the views on the possibility of its diffusion by drinking water, on the influence of conditions of soil, on the question whether the dejecta contain the poison or not, and on the duration of the incubation period. No progress was possible in combating the disease until these root questions of the etiology of cholera are decided. Hitherto the advances in knowledge upon the etiology of other infective diseases have done little towards the etiology of cholera. These advances have been made within the last ten years, during which time no opportunity—at least not in Europe—has occurred to pursue researches; and in India, where there is abundant material for such research, no one has undertaken the task. The opportunity given by the outbreak of cholera in Egypt last year to study the disease before it reached European soil was taken advantage of by various governments, who sent expeditions for the purpose. He had the honor to take part in one of these, and in accepting it he well knew the difficulties of the task before him, for hardly anything was known about the cholera poison, or where it should be sought; whether it was to be found only in the intestinal canal, or in the blood, or elsewhere. Nor was it known whether it was of bacterial nature, or fungoid, or an animal parasite—*e. g.*, an amœba. But other difficulties appeared in an unexpected direction. From the accounts given in text-books he had imagined that the cholera intestine would show very slight changes, and would be filled with a clear "rice-water" fluid. He had not fully recollected the conditions met with in post mortem examinations he had formerly made, and was therefore at first surprised to meet with quite a different state of things. For he soon found that in a large majority of cases remarkably severe lesions were present in the intestines. In other cases the changes were slighter, and eventually he met with some which, to a certain extent, corresponded with the type described in text-books. But it was some time, and after many inspections, before he was enabled to correctly interpret the varied changes met with. In spite of a most careful examination of all other organs and of the blood, nothing was found to establish the presence of an infective material, and attention was finally concentrated on the intestinal conditions. There were cases in which the lower segment of the small intestine, most marked immediately above the ileo-cæcal valve, extending thence upwards, was of a dark reddish-brown color, the mucous membrane being covered with superficial hemorrhages. In many cases the mucous membrane appeared to be superficially necrosed, and covered with diphtheritic patches. The intestinal contents in such cases were not colorless, but consisted of a sanguinolent, ichorous, putrid fluid. Other cases showed a gradual transition to a less marked change. The redness was less intense, and was in patches, whilst in others the injection was limited to the margins of the follicular and Peyerian glands, giving an appearance which is quite peculiar to cholera. In comparatively few cases were the changes so slight as to consist in a somewhat swollen and opaque condition of the superficial layers of the mucous membrane, with delicate rosy-red injection, and some prominence of the solitary follicles and Peyer's patches. In such cases the intestinal contents were colorless, but resembling meal-soup rather than rice-water. In only a solitary instance were the contents watery and mucoid. Microscopical examination of the intestine and its contents revealed, especially in the cases where the margins of Peyer's patches were reddened, a considerable invasion of bacteria, occurring partly within the tubular glands, partly between the epithelium and basement membrane, and in some parts deeper still. Then he found cases in which, besides bacteria of one definite and constant form, there were others also accumulated within and around the tubular glands, of various size, some short and thick, others very fine; and he soon concluded that he had to do here with a primary invasion of pathogenic bacilli, which, as it were, prepared the tissues for the entrance of the non-pathogenic forms, just as he had observed in the necrotic, diphtheritic changes in the intestinal mucosa and in typhoid ulcers. Passing to speak of the microscopical characters of the contents of the bowel, Dr. Koch said that owing to the sanguinolent and putrescent character of these in the cases first examined, no conclusion was arrived at for some time. Thus he found multitudes of bacteria of various kinds, rendering it impossible to distinguish any special forms, and it was not until he had examined two acute and uncomplicated cases, before hemorrhage had occurred, and where the evacuation had not decomposed, that he found more abundantly the kind of organism which had been seen so richly in the intestinal mucosa. He then proceeded to describe the characters of this bacterium. It is smaller than the tubercle bacillus, being only about half or at most two-thirds the size of the latter, but much more plump, thicker, and slightly curved. As a rule, the curve is no more than that of a comma (,) but sometimes it assumes a semi-circular shape, and he has seen it forming a double curve like an S, these two variations from the normal being suggestive of the junction of two individual bacilli. In cultures there always appears a remarkably free development of comma shaped bacilli. These bacilli often grow out to form long threads, not in the manner of anthrax bacilli, nor with a simple undulating form but assuming the shape of delicate long spirals, a corkscrew shape, reminding one very forcibly of the spirochæte of relapsing fever. Indeed, it would be difficult to distinguish the two if placed side by side. On account of this developmental change, he doubted if the cholera organism should be ranked with bacilli; it is rather a transitional form between the ba.illus and the spirillum. Possibly it is a true spirillum, portions of which appear in the comma shape, much as in other spirilla—*e. g.*, spirilla undula, which do not always form complete spirals, but consist only of more or less curved rods. The comma bacilli thrive well in meat infusion, growing in it with great rapidity. By examining, microscopically, a drop of this broth culture the bacilli are seen in active movement, swarming at the margins of the drop, interspersed with the spiral threads, which are also apparently mobile. They grow also in other fluids—*e. g.*, very abundantly in milk, without coagulating it or changing its appearance. Also in blood serum they grow very richly. Another good nutrient medium is gelatine, wherein the comma bacilli form colonies of a perfectly characteristic kind, different from those of any other form of bacteria. The colony, when very young, appears as a pale and small spot, not completely spherical as other bacterial colonies in gelatine are wont to be, but with a more or less irregular, protruding or jagged contour. It also very soon takes on a somewhat granular appearance. As the colony increases, the granular character becomes more marked, until it seems to be made up of highly refractile granules, like a mass of particles of glass. In its further growth the gelatine is liquefied in the vicinity of the colony, which at the same time sinks down deeper into the gelatine mass, and makes a small thread-like excavation in the gelatine, in the centre of which the colony appears as a small white point. This, again, is peculiar; it is never seen, at least so marked, with any other bacterium. And a similar appearance is produced when gelatine is inoculated with a pure culture of this bacillus, the gelatine liquefying at the seat of inoculation, and the small colony continually enlarging; but above it there occurs the excavated spot, like a bubble of air floating over the bacillary colony. It gives the impression that the bacillus growth not only liquefies the gelatine, but causes a rapid evaporation of the fluid so formed. Many bacteria also have the power of so liquefying gelatine with which they are inoculated, but never do they produce such an excavation with the bladder-like cavity on the surface. Another peculiarity was the slowness with which the gelatine liquefied, and the narrow limits of this liquefaction in the case of a gelatine disc. Cultures of the comma bacillus were also made in Agar-Agar jelly, which is not liquefied by them. On potato these bacilli grow like those of glanders, forming a greyish-brown layer on the surface. The comma bacilli thrive best at temperatures between 30° and 40° C., but they are not very sensitive to low temperatures, their growth not being prevented until 17° or 16° C. is reached. In this respect they agree with anthrax bacilli. Koch made an experiment to ascertain whether a very low temperature not merely checked development but killed them, and subjected the comma bacilli to a temperature of 10° C. They were then completely frozen, but yet retained vitality, growing in gelatine afterwards. Other experiments, by excluding air from the gelatine cultures, or placing them under an exhausted bell jar, or in an atmosphere of carbonic acid, went to prove that they required air and oxygen for their growth; but the deprivation did not kill them, since on removing them from these conditions they again began to grow. The growth of these bacilli is exceptionally rapid, quickly attaining its height, and after a brief stationary period as quickly terminating. The dying bacilli lose their shape, sometimes appearing shrivelled, sometimes swollen, and then staining very slightly or not at all. The special features of their vegetation are best seen when substances which also contain other forms of bacteria are taken—*e. g.*, the intestinal contents or choleraic evacuations mixed with moistened earth or linen and kept damp. The comma bacilli in these conditions multiply with great rapidity so as to far outnumber the other forms of bacteria, which at first might have been in far greater abundance. This state of affairs does not last long; in two or three days the comma bacilli began to die off, and the other bacteria to multiply. Precisely the same thing takes place in the intestine, where, after the rapid initial vegetation is over, and when exudation of blood occurs into the bowel, the comma bacilli disappear and putrefactive bacteria predominate. Whether the occurrence of putrefaction is inimical to the comma bacilli has not been proved, but from analogy it is very probable. At any rate, it is important to know this for certain, for if it be so, then the comma bacilli will not thrive in a cesspit, and then further disinfection would be unnecessary. These bacilli thrive best in fluids containing a certain amount of nutriment. Experiments have not yet shown the limits in this respect, but Koch has found them capable of growing in meat broth diluted ten times. Again, if the nutrient medium become acid in reaction their growth is checked, at least in gelatine and meat infusion; but, singularly enough, they continue to grow on the surface of boiled potato which has become acid, showing that all acids are not equally obnoxious to them. But here, as with other substances which hinder their growth, they do not kill the bacilli. Davaine has shown that iodine is a strong bactericide. He experimented with anthrax bacilli in water to which iodine was added, and the bacilli were destroyed. But practically the organisms have to be dealt with in the alkaline contents of the bowel, or in the blood or fluids of the tissues, where iodine cannot remain in the free state. Koch found that the addition of an aqueous solution of iodine (1 in 4,000) to meat infusion, in the proportion of 1 in 10, did not in the least interfere with the growth of the bacilli in that medium. He did not pursue this line of inquiry, seeing that in practice larger quantities of iodine than that could not be given. Alcohol first checks the development of the comma bacilli when it is mixed with the nutrient fluid in the proportion of 1 in 10, a degree of concentration which renders it impracticable for treatment. Common salt was added to the extent of 2 per cent., without influencing the growth of the bacilli. Sulphate of iron, in the proportion of 2 per cent., checks this growth, probably by precipitating albuminates from the fluids, and possibly also by its acid reaction; certainly it does not seem to have any specific disinfecting action—*i. e.*, in destroying the bacilli. Indeed, Koch thinks that the admixture of sulphate of iron with fæcal matter may arrest putrefaction and really remove what may be the most destructive process to the comma bacilli. Hence he would distinguish between substances which merely arrest putrefaction and those which are bactericidal; for the former may simply serve the purpose of preserving the infective virus. Among other substances which prevent the growth of the comma bacilli may be mentioned alum, in solutions of the strength of 1 in 100; camphor, 1 in 300; carbolic acid, 1 in 400; oil of peppermint, 1 in 2,000; sulphate of copper, 1 in 2,500 (a remedy much employed, but how much would really be needed merely to hinder the growth of the bacilli in the intestine!); quinine, 1 in 5,000; and sublimate, 1 in 100,000. In contrast with the foregoing measures for preventing the growth of these bacilli is the striking fact that they are readily killed by drying. This fact is proved by merely drying a small drop of material containing the bacilli on a cover-glass, and then placing this over some of the fluid on a glass slide. With anthrax bacilli vitality is retained for nearly a week; whereas, the comma bacillus appears to be killed in a very short time. Thus it was found that although vitality was retained—depending largely upon the number of bacilli—for a short time, yet withdrawal of the nutrient fluid for an hour or even less often sufficed; and it never happened that the bacilli retained vitality after a deprivation lasting twenty-four hours. These results would seem to point to the fact that the comma bacillus does not, like the organisms of anthrax and vaccinia, pass into the resting state (Daner-zustande) by drying; and if so it is one of the most important facts in the etiology of cholera. Much, however, remains to be done, especially with regard to the soiled linen of cholera patients being kept in a damp state. He found that in soiled articles, when dried for a time, varying from twenty-four hours and upwards, the comma bacilli were quite destroyed. Nor was the destruction delayed by placing choleraic excreta in or upon earth, dry or moist, or mixed with stagnant water. In gelatine cultures the comma bacilli can be cultivated for six weeks, and also in blood serum, milk and potato, where anthrax bacilli rapidly form spores. But a resting state of the comma bacilli has never been met with—a very exceptional thing in the case of bacilli, and another reason why the organism must be regarded rather as a spirillum than a bacillus, for the spirilla require only a fluid medium, and do not, like the anthrax bacilli, thrive in the dry state. It is quite unlikely that a resting state of the comma bacillus will ever be discovered; and, moreover, its absence harmonizes with our knowledge of cholera etiology.—*The Lancet.*

